# Left Pulmonary Artery Thrombosis in a Neonate with Left Lung Hypoplasia

**DOI:** 10.1155/2012/314256

**Published:** 2012-12-12

**Authors:** Matthias P. van Schendel, Douwe H. Visser, Lukas A. J. Rammeloo, Mark G. Hazekamp, Jaroslav Hruda

**Affiliations:** ^1^Department of Pediatric Cardiology, VU University Medical Center, P.O. Box 7057, 1007 MB Amsterdam, The Netherlands; ^2^Department of Neonatology, VU University Medical Center, Amsterdam, The Netherlands; ^3^Department of Cardiothoracic Surgery, Leiden University Medical Center, The Netherlands

## Abstract

Thrombotic events in neonates may origin from fetal life. A 4-day-old newborn infant with a family history of heterozygous type 1 protein C deficiency was diagnosed with left lung hypoplasia and left pulmonary artery thrombosis. Its source was prenatally closed ductus arteriosus. Surgical removal of the thrombus was performed.

## 1. Introduction

Thrombotic events in neonates have been recorded with increasing frequency, especially when an inherited thrombophilia is present [1]. In many cases the thrombotic process has started long before the delivery. We present the case of a newborn infant with pulmonary artery thrombosis (PAT) and left lung hypoplasia with a family history of heterozygous type 1 protein C deficiency. 

## 2. Case Presentation

**Figure 1:**
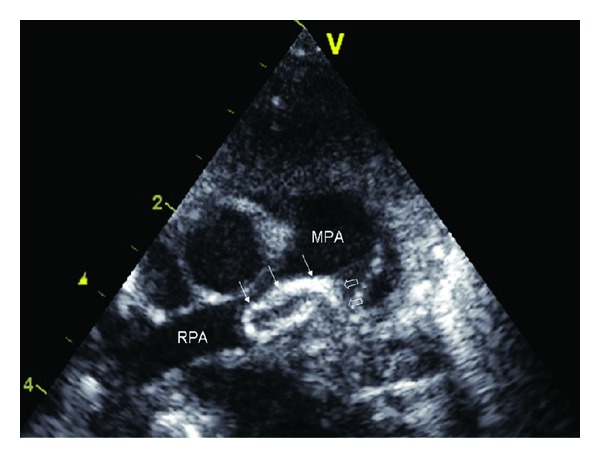
Cross-sectional echocardiogram showing the protrusion of the echodense mass (arrows) into the right pulmonary artery, extending from obstructed left pulmonary artery (block arrows). MPA: main pulmonary artery. RPA: right pulmonary artery.

A 37-week-old female baby was born via secondary cesarean section because of fetal bradycardia. Mother, a 29-years-old gravida 1, para 0, did not use medication during pregnancy. Antenatal ultrasonography showed no structural fetal abnormalities. Oligohydramnion and impaired fetal growth complicated in the last trimester. Birth weight and length were 2080 grams (−2 SD) and 42 cm (−2 SD), respectively. Short after birth she developed respiratory distress. Chest X-ray revealed diminished left lung volume and poor lung aeration. A systolic ejection murmur grade 2/6 was heard on day 4. Echocardiography showed normal intracardiac anatomy and equal aortic and pulmonary pressures. No left pulmonary artery (LPA) lumen and flow could be identified; the whole LPA lumen was completely filled with a homogeneous content with increased echogenicity. A lobular echodense mass was protruding into the main pulmonary artery, originating at the entrance of the ductus arteriosus (DA) and extending in the length of 10 millimeters into the right pulmonary artery (RPA) (Figure 1), occupying two thirds of the lumen of the RPA and causing a systolic pressure gradient of 15 mmHg. The DA was obliterated. All four pulmonary veins drained normally into the left atrium, and the pulmonary venous return from the left lung was diminished. Computed tomography angiography demonstrated a hypoplastic left lung with small cysts. The LPA did not fill with contrast, and solely the RPA was visualized. Several collateral systemic arteries were supplying the left lung. Low molecular weight heparin treatment was started in order to prevent growth of the suspected PAT. However, in the course of several days the mass further extended into the RPA, with further increase of the pressure gradient. Surgical removal of this pulmonary artery mass was performed. A white colored matter of atheromatous appearance originating from the ductal orifice filled the LPA and extended into the RPA. This white matter was removed; nevertheless the periphery of the hypoplastic LPA remained obstructed. Histologic examination identified fibrous organized calcified matter, consistent with an old thrombus originating from fetal life. Eight months after surgery, the baby remains asymptomatic and develops properly. Echocardiography demonstrated normal pulmonary venous return right and mildly reduced left, consistent with minimal prograde flow in the LPA along with some collateral flow. The RPA morphology and flow were normal. No pulmonary hypertension was detected. There was a paternal family history of heterozygous type 1 protein C deficiency. Even though protein C activity in the first days after partum reached 17% (normal range 14–42%), repeated exam at the age of six months confirmed protein C deficiency (activity 26% (normal range 70–125%), antigen 22% (normal range 70–125%). 

## 3. Discussion

The incidence of PAT is estimated 0.14–0.90 per 100.000 in the community and 8.6–57.0 per 100.000 in hospitalized children [2]. PAT was reported even in newborn infants [3–16]. In the majority of the reports PAT developed already in utero. The thrombus originated in several reports from fetal DA [4–6], similar to our case, where ductal origin of the thrombus was documented both with echocardiography and preoperatively. This entity may occasionally remain unrecognized and untreated; however, in most cases PAT presents as respiratory failure [3], severe persistent pulmonary hypertension of the newborn, or cyanotic congenital heart disease [8]. Surgical embolectomy may be required [9–11]. Predisposing factors for neonatal PAT include central venous lines, congenital heart disease and infection. However, in many cases PAT originates from fetal life. Inherited prethrombotic disorders are present in less than 10% of the pediatric cases [1, 11]. In contrary to its homozygous form, heterozygous protein C deficiency is in most patients asymptomatic, and its role in developing intrauterine PA remains purely speculative.

In our patient, surgical intervention was necessary because the PAT exhibited further growth from the bifurcation and threatened to obstruct the RPA. However, histologic examination demonstrated a white fibrous mass consistent with an old, antenatally formed thrombus, which could not have had the potential for growth. Therefore, we probably observed the passive elastic deformation and extension of this mass into the vessel, rather than the true growth. 

The association of unilateral PAT and lung hypoplasia is intriguing. It is believed that normal flow in the pulmonary artery is not necessary for adequate development of the lung in the fetus. We could find only two reports on neonatal PAT with left lung hypoplasia [13, 14]. Elhassan et al. postulated that a vascular injury in week 5–8 after gestation could be responsible for the arrest of lung maturation in utero and might be the main reason for both LPA thrombosis and left pulmonary hypoplasia [13]. Their alternative explanation was that left pulmonary vasculature and lung development were abnormal, and a thrombosis developed later in gestation [13]. The small cysts within the hypoplastic lung seen on CT in our patient were most probably postinfarction cysts [14]. Children with unilateral absence of the pulmonary artery are usually born with sufficiently developed lungs [17], but if the ipsilateral DA was closed, lung hypoplasia developed [17]; a setting resembling prenatal thrombotic DA closure causing occlusion of the LPA. In congenital diaphragmatic hernia, fetal pulmonary artery diameters correlate with lung hypoplasia [18]. Capillary embolization in fetal age impairs alveolarization [19]. The degree of pulmonary hypoplasia associated with abnormal pulmonary arterial flow depends on the timing of the insult and the amount of time allowed for the collateral circulation to develop [20]. 

In conclusion, intrauterine DA closure by a thrombus can extend into the ipsilateral pulmonary artery and may be associated with unilateral lung hypoplasia. Thrombophilic disorders such as protein C deficiency should be sought as one of the contributing factors. 

## Abbreviations

DA: Ductus arteriosus
LPA: Left pulmonary artery
PAT: Pulmonary arterial thrombosis
RPA: Right pulmonary artery.
